# Supplementary material for: Effectiveness of implementation strategies for uptake of fall prevention interventions in community care: A systematic review
Source: PLoS One. 2025 Jul 10;20(7):e0322727. doi: 10.1371/journal.pone.0322727 (PMC12244475; doi:10.1371/journal.pone.0322727)
Supplement: S2 Table — Additional file 2: Search strategy. (DOCX) [file pone.0322727.s002.docx]

# **Search strategy**

**Database**: Ovid MEDLINE(R) ALL <1946 to December 04, 2020>

**Date**: 09.12.2020

**Results**: 2024

| **#** | **Searches** | **Results** |
| --- | --- | --- |
| 1 | aged/ | 3128614 |
| 2 | "aged, 80 and over"/ | 933517 |
| 3 | frail elderly/ | 11863 |
| 4 | geriatric nursing/ | 13628 |
| 5 | geriatrics/ | 30191 |
| 6 | Health Services for the Aged/ | 17843 |
| 7 | (aged or elder* or eldest or old* or geriatric* or senior* or aging or ageing or frail or frailty).tw,kw,kf. | 2287516 |
| 8 | or/1-7 | 4801762 |
| 9 | Accidental Falls/ | 24780 |
| 10 | ((fall or falls or faller* or fallen or falling or slip or slipping) adj3 (prevent* or reduce* or reduction* or reducing* or manage*)).tw,kw,kf. | 10609 |
| 11 | (fall or falls or faller* or fallen or falling or slip or slipping).ti,kw. | 25358 |
| 12 | or/9-11 | 41896 |
| 13 | accident prevention/ | 9187 |
| 14 | safety management/ | 20358 |
| 15 | Harm Reduction/ | 3211 |
| 16 | ((fall or falls or faller* or fallen or falling or slip or slipping) adj3 (prevent* or reduce* or reduction* or reducing* or manage*)).tw,kw,kf. | 10609 |
| 17 | (prevent* or reduce* or reduction* or reducing* or manage*).ti,kw. | 1023582 |
| 18 | Prevention & Control.fs. | 1307307 |
| 19 | or/13-18 | 2087651 |
| 20 | 8 and 12 and 19 | 11398 |
| 21 | Implementation Science/ | 537 |
| 22 | Health Plan Implementation/ | 6139 |
| 23 | guideline adherence/ | 32717 |
| 24 | "outcome and process assessment, health care"/ | 27461 |
| 25 | outcome assessment, health care/ | 74159 |
| 26 | process assessment, health care/ | 4784 |
| 27 | program evaluation/ | 63908 |
| 28 | quality assurance, health care/ | 56124 |
| 29 | Program Development/ | 29375 |
| 30 | total quality management/ | 12559 |
| 31 | quality improvement/ | 26046 |
| 32 | quality indicators, health care/ | 15967 |
| 33 | "standard of care"/ | 2790 |
| 34 | "quality of health care"/ | 73682 |
| 35 | health care evaluation mechanisms/ | 0 |
| 36 | Information Dissemination/ | 17202 |
| 37 | organizational innovation/ | 24649 |
| 38 | change management/ | 110 |
| 39 | "diffusion of innovation"/ | 17788 |
| 40 | technology transfer/ | 2093 |
| 41 | Translational Medical Research/ | 11141 |
| 42 | clinical audit/ | 1779 |
| 43 | medical audit/ | 17119 |
| 44 | nursing audit/ | 3009 |
| 45 | Knowledge Management/ | 367 |
| 46 | Professional Practice Gaps/ | 182 |
| 47 | evidence-based practice/ | 10410 |
| 48 | evidence-based medicine/ | 73541 |
| 49 | evidence-based nursing/ | 3805 |
| 50 | implement*.tw,kw,kf. | 517181 |
| 51 | ((knowledge or research* or evidence*) adj2 (translat* or exchang* or transfer* or integrat* or uptak* or disseminat* or diffus* or utili* or mobili* or manage*)).tw,kw,kf. | 61585 |
| 52 | (evidence based or best practice*).tw,kw,kf. | 145367 |
| 53 | (evidence inform* adj5 (decision* or plan* or policy or policies or practice or action*)).tw,kw,kf. | 1069 |
| 54 | (adherence or change management or "sustain* use" or sustainable or sustainability).tw,kw,kf. | 202777 |
| 55 | ((chang* or improv* or effect* or influenc* or alter* or adapt* or amend* or modify* or adjust* or transform*) adj1 (policy or policies)).tw,kw,kf. | 11504 |
| 56 | (quality adj (assurance or improvement* or initiativ* or plan* or program* or review or audit or manage* or control*)).tw,kw,kf. | 122017 |
| 57 | (((behavior* or behaviour* or knowledge or organi?ational) adj1 change*) or adhere* or disseminat* or uptak* or diffus* or compliance* or changing* or audit* or feedback* or turning or capacity or mediation or transfer* or translat* or utilization or utilisation or barrier* or facilitat* or knowledge or accept* or feasibilit* or adapt* or fidelit* or "cost and return*" or sustain* or continu* or integrat* or incorporat*).ti,kf,kw. | 1428493 |
| 58 | or/21-57 | 2549037 |
| 59 | 20 and 58 | 2832 |
| 60 | limit 59 to yr="2010 -Current" | 2024 |

**Database**: Embase via Ovid <1974 to 2020 December 07>

**Date**: 09.12.2020

**Results**: 2530

| **#** | **Searches** | **Results** |
| --- | --- | --- |
| 1 | aged/ | 3067924 |
| 2 | frail elderly/ | 10417 |
| 3 | very elderly/ | 213132 |
| 4 | geriatrics/ | 30516 |
| 5 | geriatric care/ | 14809 |
| 6 | elderly care/ | 41040 |
| 7 | geriatric nursing/ | 12269 |
| 8 | (aged or elder* or eldest or old* or geriatric* or senior* or aging or ageing or frail or frailty).tw,kw. | 3167545 |
| 9 | or/1-8 | 5393656 |
| 10 | falling/ | 41845 |
| 11 | ((fall or falls or faller* or fallen or falling or slip or slipping) adj3 (prevent* or reduce* or reduction* or reducing* or manage*)).tw,kw. | 14423 |
| 12 | (fall or falls or faller* or fallen or falling or slip or slipping).ti,kw. | 33291 |
| 13 | or/10-12 | 65818 |
| 14 | prevention/ | 269124 |
| 15 | "prevention and control"/ | 26550 |
| 16 | accident prevention/ | 15510 |
| 17 | primary prevention/ | 41392 |
| 18 | secondary prevention/ | 29189 |
| 19 | tertiary prevention/ | 440 |
| 20 | harm reduction/ | 6707 |
| 21 | risk management/ | 43481 |
| 22 | ((fall or falls or faller* or fallen or falling or slip or slipping) adj3 (prevent* or reduce* or reduction* or reducing* or manage*)).tw,kw. | 14423 |
| 23 | (prevent* or reduce* or reduction* or reducing* or manage*).ti,kw. | 1367831 |
| 24 | or/14-23 | 1683011 |
| 25 | 9 and 13 and 24 | 12309 |
| 26 | implementation science/ | 1538 |
| 27 | translational research/ | 18109 |
| 28 | protocol compliance/ | 14633 |
| 29 | health care quality/ | 244793 |
| 30 | exp program evaluation/ | 26225 |
| 31 | practice gap/ | 670 |
| 32 | total quality management/ | 68400 |
| 33 | information dissemination/ | 21714 |
| 34 | change management/ | 1774 |
| 35 | mass communication/ | 14566 |
| 36 | quality control procedures/ | 1924 |
| 37 | clinical audit/ | 5135 |
| 38 | nursing audit/ | 33 |
| 39 | knowledge management/ | 1744 |
| 40 | evidence based practice/ | 67421 |
| 41 | evidence based medicine/ | 113638 |
| 42 | evidence based nursing/ | 4318 |
| 43 | evidence based practice center/ | 3611 |
| 44 | evidence-based pharmacy/ | 22 |
| 45 | patient compliance/ | 135317 |
| 46 | evaluation study/ | 47895 |
| 47 | professional standard/ | 42050 |
| 48 | program development/ | 24546 |
| 49 | implement*.tw,kw. | 687050 |
| 50 | ((knowledge or research* or evidence*) adj2 (translat* or exchang* or transfer* or integrat* or uptak* or disseminat* or diffus* or utili* or mobili* or manage*)).tw,kw. | 80452 |
| 51 | (evidence based or best practice*).tw,kw. | 199529 |
| 52 | (evidence inform* adj5 (decision* or plan* or policy or policies or practice or action*)).tw,kw. | 1239 |
| 53 | (adherence or change management or "sustain* use" or sustainable or sustainability).tw,kw. | 285348 |
| 54 | ((chang* or improv* or effect* or influenc* or alter* or adapt* or amend* or modify* or adjust* or transform*) adj1 (policy or policies)).tw,kw. | 13996 |
| 55 | (quality adj (assurance or improvement* or initiativ* or plan* or program* or review or audit or manage* or control*)).tw,kw. | 191558 |
| 56 | (((behavior* or behaviour* or knowledge or organi?ational) adj1 change*) or adhere* or disseminat* or uptak* or diffus* or compliance* or changing* or audit* or feedback* or turning or capacity or mediation or transfer* or translat* or utilization or utilisation or barrier* or facilitat* or knowledge or accept* or feasibilit* or adapt* or fidelit* or "cost and return*" or sustain* or continu* or integrat* or incorporat*).ti,kw. | 1770105 |
| 57 | or/26-56 | 3316427 |
| 58 | 25 and 57 | 3336 |
| 59 | limit 58 to yr="2010 -Current" | 2530 |

**Database:** APA PsycInfo via Ovid 1806 to November Week 5 2020

**Date**: 09.12.2020

**Results**: 428

| # | Searches | Results |
| --- | --- | --- |
| 1 | Aging/ | 59690 |
| 2 | Geriatrics/ | 11595 |
| 3 | ("380" or "390").ag. | 332578 |
| 4 | Healthy Aging/ or Aging in Place/ | 1109 |
| 5 | (aged or elder* or eldest or old* or geriatric* or senior* or aging or ageing or frail or frailty).tw. | 745675 |
| 6 | or/1-5 | 903925 |
| 7 | falls/ | 3050 |
| 8 | ((fall or falls or faller* or fallen or falling or slip or slipping) adj3 (prevent* or reduce* or reduction* or reducing* or manage*)).tw. | 2230 |
| 9 | (fall or falls or faller* or fallen or falling or slip or slipping).ti,id. | 5707 |
| 10 | or/7-9 | 6724 |
| 11 | accident prevention/ or prevention/ | 32744 |
| 12 | harm reduction/ or risk management/ | 8626 |
| 13 | ((fall or falls or faller* or fallen or falling or slip or slipping) adj3 (prevent* or reduce* or reduction* or reducing* or manage*)).tw. | 2230 |
| 14 | (prevent* or reduce* or reduction* or reducing* or manage*).ti,id. | 235177 |
| 15 | or/11-14 | 242611 |
| 16 | 6 and 10 and 15 | 1694 |
| 17 | evidence based practice/ | 18264 |
| 18 | best practices/ | 5181 |
| 19 | treatment effectiveness evaluation/ or evaluation/ or clinical audits/ | 44638 |
| 20 | "treatment process and outcome measures"/ | 68 |
| 21 | "Quality of Services"/ or "Quality of Care"/ or Professional Standards/ | 26668 |
| 22 | organizational effectiveness/ | 12400 |
| 23 | information dissemination/ | 1776 |
| 24 | Knowledge Transfer/ | 2964 |
| 25 | organizational change/ | 9938 |
| 26 | change strategies/ or "stages of change"/ | 1365 |
| 27 | technology transfer/ or knowledge management/ | 4467 |
| 28 | reporting standards/ | 212 |
| 29 | Procedural Knowledge/ | 1841 |
| 30 | Program Development/ | 7268 |
| 31 | program evaluation/ | 12779 |
| 32 | implement*.tw. | 183191 |
| 33 | ((knowledge or research* or evidence*) adj2 (translat* or exchang* or transfer* or integrat* or uptak* or disseminat* or diffus* or utili* or mobili* or manage*)).tw. | 38897 |
| 34 | (evidence based or best practice*).tw. | 62495 |
| 35 | (evidence inform* adj5 (decision* or plan* or policy or policies or practice or action*)).tw. | 440 |
| 36 | (adherence or change management or "sustain* use" or sustainable or sustainability).tw. | 52791 |
| 37 | ((chang* or improv* or effect* or influenc* or alter* or adapt* or amend* or modify* or adjust* or transform*) adj1 (policy or policies)).tw. | 7153 |
| 38 | (quality adj (assurance or improvement* or initiativ* or plan* or program* or review or audit or manage* or control*)).tw. | 11778 |
| 39 | (((behavior* or behaviour* or knowledge or organi?ational) adj1 change*) or adhere* or disseminat* or uptak* or diffus* or compliance* or changing* or audit* or feedback* or turning or capacity or mediation or transfer* or translat* or utilization or utilisation or barrier* or facilitat* or knowledge or accept* or feasibilit* or adapt* or fidelit* or "cost and return*" or sustain* or continu* or integrat* or incorporat*).ti,id. | 461633 |
| 40 | or/17-39 | 782844 |
| 41 | 16 and 40 | 569 |
| 42 | limit 41 to yr="2010 -Current" | 428 |

**Database**: Web of Science (Indexes=SCI-EXPANDED, SSCI, A&HCI, ESCI)

**Date**: 09.12.2020

**Results:**  1520

| Set | Search | Results |
| --- | --- | --- |
| # 1 | TS=(aged or elder* or eldest or old* or geriatric* or senior* or aging or ageing or frail or frailty) | 4,200,162 |
| # 2 | TS=((fall or falls or faller* or fallen or falling or slip or slipping) NEAR/2 (prevent* or reduce* or reduction* or reducing* or manage*) ) | 12,545 |
| # 3 | TI=(fall or falls or faller* or fallen or falling or slip or slipping) OR AK=(fall or falls or faller* or fallen or falling or slip or slipping) | 85,105 |
| # 4 | #3 OR #2 | 90,097 |
| # 5 | TS=((fall or falls or faller* or fallen or falling or slip or slipping) NEAR/2 (prevent* or reduce* or reduction* or reducing* or manage*) ) | 12,545 |
| # 6 | TI=(prevent* or reduce* or reduction* or reducing* or manage*) or AK=(prevent* or reduce* or reduction* or reducing* or manage*) | 1,705,469 |
| # 7 | #5 or #6 | 1,712,751 |
| # 8 | #1 and #4 and #7 | 7,527 |
| # 9 | TS=(implement*) | 1,251,592 |
| # 10 | TS=((knowledge or research* or evidence*) NEAR/1 (translat* or exchang* or transfer* or integrat* or uptak* or disseminat* or diffus* or utili* or mobili* or manage*) ) | 118,444 |
| # 11 | TS=(“evidence based” or ”best practice*”) | 161,962 |
| # 12 | TS=("evidence inform*" NEAR/4 (decision* or plan* or policy or policies or practice or action*) ) | 1,370 |
| # 13 | TS=(adherence or “change management” or “sustain* use” or sustainable or sustainability) | 506,641 |
| # 14 | TS=((chang* or improv* or effect* or influenc* or alter* or adapt* or amend* or modify* or adjust* or transform*) NEAR/1 (policy or policies) ) | 51,874 |
| # 15 | TS=(quality NEAR/0 (assurance or improvement* or initiativ* or plan* or program* or review or audit or manage* or control*) ) | 179,721 |
| # 16 | TI=(((behavior* or behaviour* or knowledge or organisational or organizational) NEAR/1 change*) or adhere* or disseminat* or uptak* or diffus* or compliance* or changing* or audit* or feedback* or turning or capacity or mediation or transfer* or translat* or utilization or utilisation or barrier* or facilitat* or knowledge or accept* or feasibilit* or adapt* or fidelit* or "cost and return*" or sustain* or continu* or integrat* or incorporat*) | 2,568,331 |
| # 17 | AK=(((behavior* or behaviour* or knowledge or organisational or organizational) NEAR/1 change*) or adhere* or disseminat* or uptak* or diffus* or compliance* or changing* or audit* or feedback* or turning or capacity or mediation or transfer* or translat* or utilization or utilisation or barrier* or facilitat* or knowledge or accept* or feasibilit* or adapt* or fidelit* or "cost and return*" or sustain* or continu* or integrat* or incorporat*) ) | 1,362,304 |
| # 18 | #17 OR #16 OR #15 OR #14 OR #13 OR #12 OR #11 OR #10 OR #9 | 4,913,641 |
| # 15 | #18 AND #8  Timespan=2010-2020 | 1,520 |

**Database**: Cochrane Library via Wiley

**Date**: 09.12.2020

**Results**: 1245

| ID | Search | Hits |
| --- | --- | --- |
| #1 | MeSH descriptor: [Aged] this term only | 206457 |
| #2 | MeSH descriptor: [Aged, 80 and over] this term only | 52584 |
| #3 | MeSH descriptor: [Frail Elderly] this term only | 719 |
| #4 | MeSH descriptor: [Geriatric Nursing] this term only | 178 |
| #5 | MeSH descriptor: [Geriatrics] this term only | 204 |
| #6 | MeSH descriptor: [Health Services for the Aged] this term only | 448 |
| #7 | (aged or elder* or eldest or old* or geriatric* or senior* or aging or ageing or frail or frailty):ti,ab,kw | 586741 |
| #8 | {OR #1-#7} | 586741 |
| #9 | MeSH descriptor: [Accidental Falls] this term only | 1488 |
| #10 | ((fall or falls or faller* or fallen or falling or slip or slipping) NEAR/3 (prevent* or reduce* or reduction* or reducing* or manage*)):ti,ab,kw | 3321 |
| #11 | (fall or falls or faller* or fallen or falling or slip or slipping):ti,kw | 4420 |
| #12 | {OR #9-#11} | 5284 |
| #13 | MeSH descriptor: [Accident Prevention] this term only | 133 |
| #14 | MeSH descriptor: [Safety Management] this term only | 172 |
| #15 | MeSH descriptor: [Harm Reduction] this term only | 126 |
| #16 | ((fall or falls or faller* or fallen or falling or slip or slipping) NEAR/3 (prevent* or reduce* or reduction* or reducing* or manage*)):ti,ab,kw | 3321 |
| #17 | (prevent* or reduce* or reduction* or reducing* or manage*):ti,kw | 272570 |
| #18 | MeSH descriptor: [] explode all trees and with qualifier(s): [prevention & control - PC] | 93092 |
| #19 | {OR #13-#18} | 273513 |
| #20 | #8 AND #12 AND #19 | 3012 |
| #21 | MeSH descriptor: [Implementation Science] this term only | 34 |
| #22 | MeSH descriptor: [Health Plan Implementation] this term only | 176 |
| #23 | MeSH descriptor: [Guideline Adherence] this term only | 1053 |
| #24 | MeSH descriptor: [Outcome and Process Assessment, Health Care] this term only | 2207 |
| #25 | MeSH descriptor: [Outcome Assessment, Health Care] this term only | 7481 |
| #26 | MeSH descriptor: [Process Assessment, Health Care] this term only | 246 |
| #27 | MeSH descriptor: [Program Evaluation] this term only | 6147 |
| #28 | MeSH descriptor: [Program Development] this term only | 710 |
| #29 | MeSH descriptor: [Quality Assurance, Health Care] this term only | 602 |
| #30 | MeSH descriptor: [Total Quality Management] this term only | 139 |
| #31 | MeSH descriptor: [Quality Improvement] this term only | 714 |
| #32 | MeSH descriptor: [Quality Indicators, Health Care] this term only | 218 |
| #33 | MeSH descriptor: [Standard of Care] this term only | 263 |
| #34 | MeSH descriptor: [Quality of Health Care] this term only | 861 |
| #35 | MeSH descriptor: [Health Care Evaluation Mechanisms] this term only | 0 |
| #36 | MeSH descriptor: [Information Dissemination] this term only | 233 |
| #37 | MeSH descriptor: [Organizational Innovation] this term only | 107 |
| #38 | MeSH descriptor: [Change Management] this term only | 2 |
| #39 | MeSH descriptor: [Diffusion of Innovation] this term only | 132 |
| #40 | MeSH descriptor: [Technology Transfer] this term only | 12 |
| #41 | MeSH descriptor: [Translational Medical Research] this term only | 116 |
| #42 | MeSH descriptor: [Clinical Audit] this term only | 23 |
| #43 | MeSH descriptor: [Medical Audit] this term only | 222 |
| #44 | MeSH descriptor: [Nursing Audit] this term only | 46 |
| #45 | MeSH descriptor: [Knowledge Management] this term only | 1 |
| #46 | MeSH descriptor: [Professional Practice Gaps] this term only | 3 |
| #47 | MeSH descriptor: [Evidence-Based Practice] this term only | 285 |
| #48 | MeSH descriptor: [Evidence-Based Medicine] this term only | 892 |
| #49 | MeSH descriptor: [Evidence-Based Nursing] this term only | 43 |
| #50 | (implement*):ti,ab,kw | 40058 |
| #51 | ((knowledge or research* or evidence*) NEAR/2 (translat* or exchang* or transfer* or integrat* or uptak* or disseminat* or diffus* or utili* or mobili* or manage*)):ti,ab,kw | 5021 |
| #52 | (evidence NEXT based or best NEXT practice*):ti,ab,kw | 19024 |
| #53 | ((evidence NEXT inform*) NEAR/5 (decision* or plan* or policy or policies or practice or action*)):ti,ab,kw | 50 |
| #54 | (adherence or change NEXT management or sustain* NEXT use or sustainable or sustainability):ti,ab,kw | 35954 |
| #55 | ((chang* or improv* or effect* or influenc* or alter* or adapt* or amend* or modify* or adjust* or transform*) NEAR/1 (policy or policies)):ti,ab,kw | 390 |
| #56 | (quality NEXT (assurance or improvement* or initiativ* or plan* or program* or review or audit or manage* or control*)):ti,ab,kw | 7887 |
| #57 | (((behavior* or behaviour* or knowledge or organisational or organizational) NEAR/1 change*) or adhere* or disseminat* or uptak* or diffus* or compliance* or changing* or audit* or feedback* or turning or capacity or mediation or transfer* or translat* or utilization or utilisation or barrier* or facilitat* or knowledge or accept* or feasibilit* or adapt* or fidelit* or "cost and return*" or sustain* or continu* or integrat* or incorporat*):ti,ab,kw | 484128 |
| #58 | {OR #21-#57} | 512660 |
| #59 | #20 AND #58 with Cochrane Library publication date Between Jan 2010 and Dec 2020, in Cochrane Reviews | 15 |
| #60 | #20 AND #58 with Publication Year from 2010 to 2020, in Trials | 1230 |
| #61 | #59 or #60 | 1245 |

**Database**: Cinahl via EBSCOhost

**Date**: 09.12.2020

**Results**: 1997

| # | Query | Results |
| --- | --- | --- |
| S1 | (MH "Aged") or (MH "Aged, 80 and Over") or (MH "Frail Elderly") or (MH "Geriatrics") or (MH "Gerontologic Nursing") | 854,795 |
| S2 | (MH "Health Services for the Aged") | 6,676 |
| S3 | TI ( (aged or elder* or eldest or old* or geriatric* or senior* or aging or ageing or frail or frailty) ) OR AB ( (aged or elder* or eldest or old* or geriatric* or senior* or aging or ageing or frail or frailty) ) | 553,640 |
| S4 | S1 OR S2 OR S3 | 1,167,939 |
| S5 | (MH "Accidental Falls") | 23,269 |
| S6 | TI ( ((fall or falls or faller* or fallen or falling or slip or slipping) N2 (prevent* or reduce* or reduction* or reducing* or manage*)) ) OR AB ( ((fall or falls or faller* or fallen or falling or slip or slipping) N2 (prevent* or reduce* or reduction* or reducing* or manage*)) ) | 7,447 |
| S7 | TI (fall or falls or faller* or fallen or falling or slip or slipping) | 18,707 |
| S8 | S5 OR S6 OR S7 | 31,510 |
| S9 | (MH "Preventive Health Care") | 20,714 |
| S10 | (MH "Safety") OR (MH "Home Safety") OR (MH "Harm Reduction") | 33,440 |
| S11 | TI ( ((fall or falls or faller* or fallen or falling or slip or slipping) N2 (prevent* or reduce* or reduction* or reducing* or manage*)) ) OR AB ( ((fall or falls or faller* or fallen or falling or slip or slipping) N2 (prevent* or reduce* or reduction* or reducing* or manage*)) ) | 7,447 |
| S12 | TI (prevent* or reduce* or reduction* or reducing* or manage*) | 383,434 |
| S13 | S9 OR S10 OR S11 OR S12 | 429,037 |
| S14 | S4 AND S8 AND S13 | 6,073 |
| S15 | (MH "Systems Implementation") OR (MH "Program Implementation") OR (MH "Implementation Science") | 29,857 |
| S16 | (MH "Guideline Adherence") | 15,548 |
| S17 | (MH "Outcome Assessment") | 44,519 |
| S18 | (MH "Process Assessment (Health Care)") OR (MH "Variance Analysis") | 7,028 |
| S19 | (MH "Program Development") | 26,511 |
| S20 | (MH "Quality Assurance") OR (MH "Quality Assessment") OR (MH "Nursing Audit") OR (MH "Process Assessment (Health Care)") OR (MH "Program Evaluation") OR (MH "Quality Improvement") OR (MH "Root Cause Analysis") OR (MH "Quality Management, Organizational") | 127,832 |
| S21 | (MH "Clinical Indicators") | 12,539 |
| S22 | (MH "Quality of Health Care") OR (MH "Professional Compliance") OR (MH "Quality of Nursing Care") | 96,459 |
| S23 | (MH "Organizational Change") OR (MH "Diffusion of Innovation") | 27,778 |
| S24 | (MH "Change Management") OR (MH "Behavioral Changes") | 17,620 |
| S25 | (MH "Knowledge Management") OR (MH "Clinical Governance") OR (MH "Clinical Effectiveness") | 5,974 |
| S26 | (MH "Professional Practice, Evidence-Based") OR (MH "Occupational Therapy Practice, Evidence-Based") OR (MH "Nursing Practice, Evidence-Based") OR (MH "Medical Practice, Evidence-Based") | 71,966 |
| S27 | TI implement* OR AB implement* | 196,703 |
| S28 | TI ( ((knowledge or research* or evidence*) N1 (translat* or exchang* or transfer* or integrat* or uptak* or disseminat* or diffus* or utili* or mobili* or manage*)) ) OR AB ( ((knowledge or research* or evidence*) N1 (translat* or exchang* or transfer* or integrat* or uptak* or disseminat* or diffus* or utili* or mobili* or manage*)) ) | 26,336 |
| S29 | TI ( "evidence based" or "best practice*" ) OR AB ( "evidence based" or "best practice*" ) | 90,744 |
| S30 | TI ( ("evidence inform*" N4 (decision* or plan* or policy or policies or practice or action*)) ) OR AB ( ("evidence inform*" N4 (decision* or plan* or policy or policies or practice or action*)) ) | 762 |
| S31 | TI ( (adherence or "change management" or "sustain* use" or sustainable or sustainability) ) OR AB ( (adherence or "change management" or "sustain* use" or sustainable or sustainability) ) | 70,201 |
| S32 | TI ( ((chang* or improv* or effect* or influenc* or alter* or adapt* or amend* or modify* or adjust* or transform*) N0 (policy or policies)) ) OR AB ( ((chang* or improv* or effect* or influenc* or alter* or adapt* or amend* or modify* or adjust* or transform*) N0 (policy or policies)) ) | 6,603 |
| S33 | TI ( (quality N0 (assurance or improvement* or initiativ* or plan* or program* or review or audit or manage* or control*)) ) OR AB ( (quality N0 (assurance or improvement* or initiativ* or plan* or program* or review or audit or manage* or control*)) ) | 38,765 |
| S34 | TI ( (((behavior* or behaviour* or knowledge or organi*ational) N0 change*) or adhere* or disseminat* or uptak* or diffus* or compliance* or changing* or audit* or feedback* or turning or capacity or mediation or transfer* or translat* or utilization or utilisation or barrier* or facilitat* or knowledge or accept* or feasibilit* or adapt* or fidelit* or "cost and return*" or sustain* or continu* or integrat* or incorporat*) ) OR AB ( (((behavior* or behaviour* or knowledge or organi*ational) N0 change*) or adhere* or disseminat* or uptak* or diffus* or compliance* or changing* or audit* or feedback* or turning or capacity or mediation or transfer* or translat* or utilization or utilisation or barrier* or facilitat* or knowledge or accept* or feasibilit* or adapt* or fidelit* or "cost and return*" or sustain* or continu* or integrat* or incorporat*) ) | 1,324,743 |
| S35 | S15 OR S16 OR S17 OR S18 OR S19 OR S20 OR S21 OR S22 OR S23 OR S24 OR S25 OR S26 OR S27 OR S28 OR S29 OR S30 OR S31 OR S32 OR S33 OR S34 | 1,699,944 |
| S36 | S14 AND S35 | 2,784 |
| S37 | S14 AND S35  Limiters - Published Date: 20100101-20201231 | 1,997 |

**Database**: Google Scholar via Publish or Perish

**Date**: 09.12.2020

**Results**: 300

implement|implementation|implementing fall|falling|faller|fallen|slip prevent|prevention|preventing|reduce|manage elderly|older|senior|aging|frail
