# Supplementary material for: Effectiveness of implementation strategies for uptake of fall prevention interventions in community care: A systematic review
Source: PLoS One. 2025 Jul 10;20(7):e0322727. doi: 10.1371/journal.pone.0322727 (PMC12244475; doi:10.1371/journal.pone.0322727)
Supplement: Attachment — Submitted filename: 12.01.25 Response to Reviewers.docx [file pone.0322727.s005.docx]

Dear Dr Rehana Abdus Salam

Thank you for considering our manuscript and allowing us to resubmit it. You and the reviewer have done an excellent job in evaluating our work, and we acknowledge the importance of the issues regarding the date of the search. We have updated the search and screened and reviewed the eligibility of 1196 new references. The update of the search has been included in the changes to the manuscript and in Figure 1.

For the additional comments, we have provided a “Point-by-Point” response. We have included a numbered quotation from you and reviewer 1, followed by our response, labeled “AR” for “Author Response” along with a corresponding numerical identifier (e.g., AR3). The responses include references to line numbers within the manuscript where changes have been made.

We believe we have adequately addressed the concerns and suggestions raised by the reviewer. The revised manuscript, including all changes from the original submission, is marked using track changes.

On behalf of all the authors, I would like to express our gratitude for considering this article, and we look forward to your response.

Sincerely,

Siv Linnerud

PhD Student

OsloMet – Oslo Metropolitan University

**Point-by-point response**

**Additional comments from Editor:**

**Q1_E: Line 130: "In case of disagreement...."**

AR1: Thank you for bringing this to our attention. The error has now been corrected; please see changes in line 132.

**Q2_E: The authors have used the terms 'recommendations' and 'interventions interchangeably and I would suggest consistency for readers.**

AR2: Thank you for addressing this. We agree that this could be confusing and have made changes to use only the term “interventions”. Please see changes in line 76, 86, 268 and 299.

**Q3_E: This statement in the Discussion section is not clear: "the use of an implementation strategy did not provide unambiguous effects for falls leading to referral to acute care."**

AR3: Thank you for addressing this. We agree that this statement is not clear to the readers. We have changed the section to “However, the use of implementation strategies did not consistently reduce the number of falls leading to referral to acute care”. Please see lines 272-274.

**Q4_E. I think that it is important to specify how the authors eligibility criteria differed from the review by Vandervelde et al in the discussion section where they state: "Vandervelde et al. (14) aimed at summarize implementation strategies in multifactorial fall prevention interventions and had other eligibility criteria than used in this current systematic review." especially when they have included three RCTs and three non_RCTs that do not overlap with the included studies in this review.**

AR4: Thank you for addressing this issue. We agree that it is important to clarify how our review differs from the review by Vandervelde et al. in terms of eligibility criteria. We have added more details on this distinction in the discussion section, specifically on lines 283-286.

**Q5_E: Line 319: Change Prospero to PROSPERO**

AR5: Thank you for bringing this to our attention. We have changed it to PROSPERO in line 331.

**Comments from Reviewer 1:**

**Q6_R1: Line 143: was the implementation strategy extracted verbatim? see comment on Table 1 below.**

AR6: Thank you for addressing this important issue. The implementation strategies were transcribed verbatim into the extraction form. However, to enhance clarity and comprehension, they have been paraphrased by the authors in the manuscript. Please see lines 146-149 and further comments in AR8.

**Q7_R1: Lines 202 – 214: I find this section difficult to follow. Suggest “the strategy used by…” rather than “ the first strategy”, or add some more context. Were the studies comparing the effects between the standard and enhanced support? In line with Table 1, suggest describing Ford et al’s study before the others.**

AR7: Thank you for highlighting these difficulties in the manuscript. We have reordered the description of the implementation strategies in the text (see lines 207-210) and arranged them in alphabetical order in the Table. The first and second implementation strategies refer to a study where two different strategies were tested in parallel. To reduce confusion, we have clarified that the two implementation strategies were tested within the same study (line 212).

**Q8_R1: Table 1: A number of changes may help readability here. Can I suggest to add reference numbers to the studies listed; I initially read Guse 2015 and Paterson 2015 as one report – two citation numbers would help here. Add “1st” to Author. Explain BHAS and ALCOVE abbreviations. Is “cluster of single strategies” described in the text? What guided your categorization of these into A-F? I would be inclined to add more detail to the interventions in the table.**

AR8: Thank you for your valuable suggestions to improve the readability of the table. We have added the first author to the headings and included citation numbers for each report. Abbreviations for BAHS and ACOVE prime have also been added to the table. Additionally, we have provided more information on the categorization of individual strategies within each implementation strategy and their clustering in the methods section, as detailed in lines 146-149.

**Q9_R1: There is a different font used in Table 1 for “comparison”.**

AR9: Thank you for bringing this to our attention, the font in Table 1 has been corrected.

**Q10_R1: Line 130 [I]n cases of disagreement….**

AR10: Thank you for bringing this to our attention. The error has now been corrected; please see changes in line 132.

**Q11_R1: Figure 1 was not clear in the PDF document.**

AR10: Thank you for pointing this out. Figure 1 is now updated and inserted in the PDF.
