# Supplementary material for: Effectiveness of implementation strategies for uptake of fall prevention interventions in community care: A systematic review
Source: PLoS One. 2025 Jul 10;20(7):e0322727. doi: 10.1371/journal.pone.0322727 (PMC12244475; doi:10.1371/journal.pone.0322727)
Supplement: Attachment — Submitted filename: 12.03.25 Respond to reviewer.docx [file pone.0322727.s006.docx]

09. March 2025

Dear Editor

Thank you for considering our manuscript. We are grateful for your constructive feedback and the opportunity to revise our manuscript. We acknowledge the important issues raised and have responded to each comment in detail below, labeled AR1, AR2, etc.

We believe we have responded adequately to all your concerns and suggestions. The new version of the manuscript, with changes from the original submission, is indicated by use of track changes. On behalf of all the authors, I would like to thank you for considering this article and look forward to your reply.

Sincerely,

Siv Linnerud

PhD Student

Department of Rehabilitation Science and Health Technology,

Oslo Metropolitan University (OsloMet), Oslo, Norway

Response to editor comments

**I would like to thank the authors for revising the draft. However, the manuscript still lacks clarity with regards to the eligibility criteria. This could be due to the fact that the authors had to ammend their protcol due to a recent publication in the same domain. However, the methodology appears to lack the rigor and I have highlighted quite a few major revisions required in order to consider the draft again for publication:

ABSTRACT:
1. Use of term 'clinical' in relation to the interventions is a bit confusing considering that the review includes studies conducted in community care rather than clinical or hospital settings. Use of the term 'clinical' in relation to the outcomes is fine since the those outcomes refer to ER admissions or hospitalisations. I would suggest if the authors could reconsider using the term 'clinical' in relation to the interventions in the abstract as well as elsewhere in the draft.**

AR1: Thank you for this important comment. We have considered the use of the term “clinical” in relation to interventions and have addressed this by removing the wording from the abstract and manuscript. **INTRODUCTION:
The introduction is lengthy and fails to make a good enough case for evaluating implementation strategies for interventions preventing falls in community settings. I have made a few suggestions below to improve that:

1. In line with the first comment under 'ABSTRACT', there is a lot of dicussion around uptake of implementation strategies in clinical practice. However, the authors do not make a good enough case for current practice and uptake in commuity settings.**

AR1: Thank you for addressing this lack in the introduction. We have addressed current practice and uptake in community setting on page 4, lines 57-62.

**2. The third paragraph in the 'Introduction' section appears to be more of a 'Discussion' around similarities and differences between this review and other existing reviews. I would suggest that the authors either move it to discussion or completely remove it if the same references are already being discussed in the 'Discussion' section.**

AR2: Thank you for pointing this out. We have suggested removing the section from the introduction. Please see page 5, lines 82-92.

**3. Paragraph 4 seems repetitive of what has already been established in the end of paragraph 2. For e.g. "When implementing clinical guidelines in general, previous systematic reviews have identified clinical decision support, practice visits, audit and feedback, use of local opinion leaders, tailored interventions, and training as effective strategies. In addition, commonly used strategies for fall prevention include the involvement of stakeholders and tailoring. However, none of these reviews have evaluated the effectiveness of these implementation strategies specifically for fall prevention." can be deleted.**

AR3: Thank you for pointing this out. We have suggested removing the suggested section from the introduction. Please see page 5, lines 93-98.  **METHODS:
1. Please state all the outcomes considered under 'Implementation Outcomes' and 'Clinical Outcomes' separately. Later in the results section, authors mention the Proctors’ taxonomy of implementation outcomes. I would suggest clarifying in the methodology if these were considered or removing them from the Results section.**

AR1: Thank you. We have added all the outcomes considered in the text describing eligibility criteria. Please see page 7, lines 125-129. Additionally, we have removed the mention of Proctors taxonomy of implementation outcomes from the Results section, please see page 12, lines 209-210.

**2. Considering that the authors have focused on the effectiveness of implementation strategies rather than interventions, I feel that it merits some elaboration in the methods section. For e.g. under the section on 'data extraction' when authors state 'details of implementation strategy' some details can be added on what implementation strategies were anticipated? which implementation strategies did author consider eligible for inclusion? Moreover, the statements like 'single strategies within each identified implementation strategy' could also be very confusing to readers who are not implementation scientists and hence some description of ERIC implementation strategies would be helpful to provide some insight on what are implementation strategies and what was eligible for inclusion in this review. The authors can also add a supplementary table for further reading here.**

AR2: We appreciate your feedback regarding the limitations in our methods section. We have expanded the 'Data Extraction' section to include detailed information on expected implementation strategies, clarifying our inclusion criteria. A table outlining these strategies has been added. See changes on page 8, lines 150-158. Further, to enhance clarity, we have differentiated between single implementation strategies, henceforth referred to as implementation strategies, and broader implementation interventions that encompass a combination of strategies throughout the manuscript.

**RESULTS:
1. Not sure why the authors have referred to the Proctors’ taxonomy of implementation outcomes under the section 'Implementation strategy' as it was not discussed in the methods section (as I have highlighted earlier). I would suggest that the authors either add the details in the methods section if any data was extracted based on Proctors' implementation outcomes or delete this statement from the Results section.**

AR1: Thank you for addressing this aspect. We have suggested removing the sentence referring to Proctors taxonomy of implementation outcomes. Please see page 12, lines 209-210.

**2. If the implementation strategies are highlighted earlier in the methods section as suggested, it would be easier for the readers to comprehend the results detailed under the section reporting on 'implementation strategies'.**

AR2: Thank you for highlighting this point. We have expanded the description of implementation strategies in the methodology section, as detailed in the AR2 under the 'Methods' subheading.

**3. Again, the statement "The second implementation strategy, known as the standard support, comprised funding and a training compendium, similar to the enhanced support (24, 28). For the purpose of this review, only the enhanced strategy has been included." does not make sense since the eligilibity of implementation strategies were not detailed in the methods section. I would suggest that the authors clearly define the implementation strategies considered for inclusion in the 'methods' section in order to guide the 'Results.**

AR3:Thank you for pointing this out. We now see that the way it is written could be misunderstood. We did not select implementation strategies based on predefined definitions; rather we included all the strategies from the included studies. We have rewritten the information from Guse and in the manuscript, and we hope it is clearer now. See page 13 Line 238-240.

**4. I would suggest that the authors separately report the implementation outcomes and the clinical outcomes in the text as well as they have reported in the table.**

AR4: Thank you for pointing this out. We believe the implementation and clinical outcomes are reported separately in the text. To further clarify, we have now created distinct paragraphs for each category. This change is reflected on page 13 and 14, lines 246-247.
